# Arsenic exposure and measures of glucose tolerance in Bangladeshi adults: A cross-sectional study

**DOI:** 10.1097/EE9.0000000000000330

**Published:** 2024-08-21

**Authors:** Maitreyi Mazumdar, Xingyan Wang, Subrata K. Biswas, Partha Pratim Biswas, Afifah Farooque, Mi-Sun Lee, Crystal M. North, Sakila Afroz, Nazmul Husain, Fuadul Islam, Md Golam Mostofa, Sadia Mow, Liming Liang, Christopher Hug, David S. Ludwig, Quazi Quamruzzaman, Abby F. Fleisch, David C. Christiani

**Affiliations:** a Department of Environmental Health, Harvard T.H. Chan School of Public Health, Boston, Massachusetts; b Department of Neurology, Boston Children’s Hospital, Boston, Massachusetts; c Department of Epidemiology, Harvard T.H. Chan School of Public Health, Boston, Massachusetts; d Department of Molecular and Cell Biology, University of Connecticut, Storrs, Connecticut; e Department of Biochemistry, Bangabandhu Sheikh Mujib Medical University, Dhaka, Bangladesh; f Division of Pulmonary and Critical Care Medicine, Massachusetts General Hospital, Boston, Massachusetts; g Dhaka Community Hospital Trust, Dhaka, Bangladesh; h Consultant, Brookline, Massachusetts; i New Balance Foundation Obesity Prevention Center, Boston Children’s Hospital, Boston, Massachusetts; j Center for Interdisciplinary Population & Health Research, MaineHealth, Westbrook, Maine; k Pediatric Endocrinology and Diabetes, Maine Medical Center, Portland, Maine

**Keywords:** Bangladesh, Arsenic, Heavy metals, Diabetes, Environmental Health, Glucose, Glucose tolerance, Exposure science

## Abstract

**Background:**

Arsenic has been associated with diabetes and impaired glucose tolerance in many studies, although some reports have shown null findings.

**Methods:**

We conducted a cross-sectional study among 300 adults in Bangladesh. Participants were randomly selected from a roster of 1800 people who previously participated in studies of arsenic and skin lesions. We measured fasting glucose and insulin levels. We assessed drinking water arsenic concentration using graphite furnace atomic absorption spectrophotometry (GF-AAS) and toenail arsenic concentration using inductively coupled mass spectrometry (ICP-MS). We ran covariant-adjusted, linear regression and spline models to examine associations of arsenic concentrations with the homeostatic model assessment of insulin resistance (HOMA-IR), a marker of insulin resistance, and HOMA of beta-cell function (HOMA-β), a marker of beta-cell function.

**Results:**

Among 285 participants with complete data, the median (IQR) arsenic concentration was 4.0 (6.9) μg/g in toenails and 39.0 (188.5) μg/L in drinking water. Arsenic concentrations were not associated with insulin resistance or beta-cell function. HOMA-IR was 0.67% lower and HOMA-β was 0.28% lower per µg/g increment in toenail arsenic, but these effect estimates were small, and confidence intervals crossed the null value.

**Conclusions:**

Although arsenic exposure has been associated with diabetes, we found no evidence of an adverse effect on insulin resistance or beta-cell function.

**What this study adds**

In a cross-sectional study of Bangladeshi adults with moderate arsenic exposure, we found no evidence for adverse effects of arsenic on insulin resistance or beta-cell function.

## Introduction

Up to 220 million people in as many as 70 countries are exposed to elevated arsenic levels through contaminated groundwater.^1,2^ Arsenic in drinking water has been associated with an increased risk of a wide range of health outcomes, including lung and skin cancer, cardiovascular disease, and renal necrosis.^3^ Arsenic exposure has also been associated with diabetes and impaired glucose tolerance in many studies,^4–7^ with the strongest evidence coming from regions where drinking water arsenic concentrations are high (i.e., greater than 300 µg/L).^8–12^ Studies conducted in populations with low to moderate levels of exposure have also demonstrated an increased incidence of diabetes with chronic arsenic exposure,^13–15^ although some reports have shown null findings.^16–18^ A recent review concluded that the evidence supports an association between arsenic and diabetes but cautioned that a major limitation includes selective reporting of results.^4^ In this study, we present an analysis of arsenic exposure, measured in toenail samples and drinking water, and insulin resistance and beta-cell function in an arsenic-exposed population of adults with moderate arsenic exposure in Bangladesh.

## Methods

### Study participants and questionnaires

Data collection took place between May 2018 and November 2022. All protocols were reviewed and approved by the institutional review boards of Boston Children’s Hospital (BCH) (Protocol number: IRB-P00024501), the Dhaka Community Hospital Trust (DCH), and the Bangabandhu Sheikh Mujib Medical University (BSMMU) (Protocol number: BSMMU/2019/3739). The Harvard T.H. Chan School of Public Health (HSPH) relied on BCH IRB review. All participants provided written consent before participation.

We enrolled 300 adults from a total of 1800 adults who participated in studies of arsenic and skin lesions between 2001 and 2003 in Bangladesh.^19^ We planned a sample size of 300, and all of the participants in the 2001–2003 study were considered eligible for inclusion in the current study. We placed potential participants onto lists using random digit assignment and contacted each potential participant in order on the list. Trained staff collected information on participant age, education, employment, smoking, and medication histories, and measured participant height and weight.

### Exposure Assessment

The Environmental Engineering Laboratory at the Bangladesh University of Engineering and Technology measured arsenic in drinking water using graphite furnace atomic absorption spectrometry.^20^ Ten percent of the samples had a water arsenic concentration below the limit of detection (LOD) of 1 µg/L. For samples below the LOD, we estimated water arsenic concentrations to be LOD/2. The Dartmouth Trace Element core facility measured arsenic in toenails using inductively coupled plasma mass spectrometry (ICP-MS).^21^ All toenail arsenic values were above the LOD of 0.002 µg/g.

### Measures of Glucose Tolerance

We collected blood samples in gold-top serum separator tubes after fasting for at least 8 hours. The Clinical Biochemistry laboratory of BSMMU measured insulin concentrations in serum using a chemiluminescent immunoassay and glucose concentrations with an enzymatic method on an ARCHITECT plus Ci4100 instrument (Abbott). We estimated insulin resistance by calculating the homeostatic model assessment of insulin resistance (HOMA-IR) as ([fasting glucose (mmol/L) × fasting insulin (µU/mL)]/22.5), and we estimated beta-cell function by calculating the homeostatic model assessment of beta-cell function (HOMA-β) as (20 × fasting insulin [µU/mL])/(fasting glucose [mmol/L]−3.5).^22^

### Statistical Analysis

We employed linear regression models to examine the associations of arsenic concentrations with HOMA-IR and HOMA-β. Due to the lognormal distributions of HOMA-IR and HOMA-β, these variables were log transferred before analysis. We fit both unadjusted and covariate-adjusted models to examine the overall relationship of arsenic in toenails (primary analysis) and in water (secondary analysis) with HOMA-IR and HOMA-β. Covariates included age (continuous), sex, body mass index (continuous), smoking status (ever vs. never), education level (illiterate/able to write vs. primary education and above), and occupation (agricultural vs. nonagricultural). We chose covariates based on the review of the literature. We fit a model adjusted for all covariates. tested each covariate individually, and reviewed the change in estimate. We exponentiated the beta coefficients and reported the results as a percent change (% change = [exp(beta)-1] X100). In sensitivity analyses, we excluded participants who reported taking hypoglycemic medications, such as metformin.

Additionally, we fit unadjusted and covariate-adjusted spline generalized additive models (GAM, R package: mgvc, V1.9-0) to examine potential nonlinear associations of arsenic concentrations with HOMA-IR and HOMA-β. We used R 4.3.2 (Eye Holes) for all analyses.

We calculated a post hoc power analysis using simulations. In each simulation, we generated a distribution of toenail arsenic concentrations for 300 samples that was based on the mean and standard deviation observed in our study. We calculated the effect of arsenic for each sample by multiplying the simulated arsenic level and effect sizes derived from our linear regression models. Random errors were then generated from a normal distribution with a mean of 0 and a variance equal to the difference between the outcome variance estimated from our data and the variance of the simulated arsenic effects. The simulated outcomes were generated by adding the arsenic effects and random errors. We fit a linear regression model without intercept to test the association with arsenic levels using a *P*-value threshold of 0.05. We conducted 300 simulations for each model. Power was calculated as the proportion of simulations where the *P* value was less than 0.05, divided by 300.

## Results

Of the 300 participants enrolled, one did not undergo blood collection, 10 were excluded because of missing toenail samples, and four were excluded for missing insulin values, leaving 285 in the analytic sample. We present participant characteristics in Table 1. No participant reported a diagnosis of diabetes in 2018–2022, but 16 (5.6%) of the 285 participants reported use of prescribed oral hypoglycemic medications. Median (IQR) toenail arsenic concentration was 4.0 (6.9) μg/g and drinking water arsenic concentration was 39.0 (188.5) μg/L (Table 2). Toenail and drinking water arsenic concentrations were moderately correlated (Spearman’s r = 0.69).

**Table 1.** Demographics of study population (n = 285).

	Total (n = 285) n (%^a^), mean ± SD or median [IQR]	Men (n = 156) n (%^a^), mean ± SD or median [IQR]	Women (n = 129) n (%^a^), mean ± SD or median [IQR]
Age (years)	51.7 ± 10.6	51.3 ± 11.7	52.2 ± 9.1
BMI (kg/m^2^)	22.8 ± 3.7	22.5 ± 3.6	23.1 ± 3.8
Education			
Able to write	109 (38.2)	41 (26.3)	68 (52.7)
Primary education or above	176 (61.8)	115 (73.7)	61 (47.3)
Occupation			
Agricultural	69 (24.2)	68 (44.2)	-
Nonagricultural	216 (75.8)	87 (55.8)	129 (100.0)
Smoking status			
Ever	86 (30.2)	86 (55.1)	-
Never	199 (69.8)	70 (44.9)	129 (100.0)
Hypoglycemic medications	16 (5.6)	8 (5.1)	8 (6.2)
HOMA-IR	1.2 (1.4)	1.1 (1.5)	1.3 (1.3)
HOMA-β	85.6 (95.0)	80.0 (86.8)	90.9 (115.1)

a Column sums of individual percentages may not total 100 due to rounding.

**Table 2.** Toenail and water arsenic (As) concentration values (n =285)

	n	25th Percentile	50th Percentile	75th Percentile	Min	Max	IQR
Toenail as concentration, μg/g	285	1.3	4.0	8.2	0.2	31.6	6.9
Water as concentration, μg/L	275	8.0	39.0	196.5	0.5	1239.0	188.5

In unadjusted models, we found each unit increment in toenail arsenic concentration to be associated with a 2.16% lower HOMA-IR (β =−0.0218; 95% CI = −0.0296, −0.0140) and 0.70% lower HOMA-β (β = −0.0070; 95% CI = −0.7498, 0.7358) In models adjusted for all covariates, toenail and water arsenic concentrations were not associated with HOMA-IR or HOMA-β (Table 3). When we tested covariates individually, education level was the only variable associated with the change in estimates. Models that excluded participants taking hypoglycemic medications showed similar results (data not shown). Generalized additive models suggested a linear relationship of arsenic measures with HOMA-IR or HOMA-β (data not shown).

**Table 3.** Linear regression results of unadjusted and covariate-adjusted^a^ models

Outcomes	Predictors	Unadjusted models *β* (95% CI)	Covariate-adjusted models *β* (95% CI)
ln (HOMA-IR)	Toenail arsenic	−0.0218 (−0.0296, −0.0140)	−0.0067 (−0.5712, 0.5578)
	Water arsenic	−0.00027 (−0.4746, 0.4740)	−0.000018 (−1.8091, 1.8090)
ln (HOMA-β)	Toenail arsenic	−0.0070 (−0.7498, 0.7358)	−0.0028 (−1.4140, 1.4084)
	Water arsenic	0.000097 (−1.3621, 1.3623)	0.00011 (−1.2601, 1.2604)

a Covariates adjusted are age, sex, education, occupation, smoking status, and BMI.

BMI indicates body mass index.

The estimated power for arsenic and HOMA-IR analysis was 0.987 and 0.273 for unadjusted and adjusted models, respectively. For the arsenic and HOMA-β analysis, the estimated power was 0.31 and 0.08 for the unadjusted and adjusted models, respectively. These findings align with our analysis, indicating that the unadjusted model assessing arsenic and HOMA-IR is statistically significant, while all other models are not.

## Discussion

We found no association between arsenic exposure and insulin resistance (HOMA-IR) or beta-cell function (HOMA-β) in a cross-sectional study of Bangladeshi adults with moderate arsenic exposure.

While increasing evidence links arsenic exposure to diabetes,^4–7^ studies with positive associations most often use either a clinical diagnosis of diabetes^6–8^ or elevated hemoglobin A1c^11,23^ as outcome measures. Studies that use direct biochemical measures of insulin and glucose may be better positioned to detect more subtle effects of arsenic exposure on insulin resistance and insulin secretion. Most of the studies that measure insulin and glucose have found no association between arsenic and HOMA-IR,^23–26^ although a recent study conducted in an area of Bangladesh with higher levels of arsenic demonstrated positive associations between water, hair, and toenail arsenic concentrations and HOMA-IR.^10^ In that study, HOMA-IR levels in a high arsenic group were 2.5-fold greater than those in the low exposure groups, and comparison of HOMA-IR levels between moderate and low exposure groups showed no significant differences. Although there was a wide range of arsenic exposure in our population (<LOD to 1239.0 µg/L), most of our study population had low and moderate exposures (<300 µg/L). Our null finding might be a result of a dose-dependent effect of arsenic on measures of glucose tolerance.

A strength of our study is the measurement of arsenic in toenails, a biomarker that reflects long-term internal exposure.^27^ Many previous studies measured arsenic in spot urine samples,^23–25,28^ raising concerns that the null findings observed in those studies were a result of exposure misclassification. The use of toenails provides a more integrated, long-term exposure measure than urine.^29^

Our study has several limitations. First, the study was cross-sectional, and therefore we are not able to assess critical windows of exposure that may be important.^30^ Limitations also include low power due to the relatively small sample size and few highly exposed individuals.

## Conclusions

In a cross-sectional study of Bangladeshi adults with moderate arsenic exposure, we found no evidence for adverse effects of arsenic on insulin resistance or beta-cell function.

## Conflicts of interest statement

The authors declare that they have no conflicts of interest with regard to the content of this report.

## ACKNOWLEDGMENTS

We thank Shaye Moore for her review of the manuscript.
